# Role of MicroRNAs in Cardiac Disease with Stroke in Pregnancy

**DOI:** 10.1155/2022/5260085

**Published:** 2022-09-12

**Authors:** Umme Salma, Ahmed Baker A. Alshaikh, Muhannad Faleh Alanazi, Basil Mohammed Alomair, Mubarak Alruwaili, Raed Alruwaili

**Affiliations:** ^1^Department of Obstetrics & Gynecology, College of Medicine, Jouf University, Sakaka, Saudi Arabia; ^2^Department of Internal Medicine, Division of Radiology, College of Medicine, Jouf University, Sakaka, Saudi Arabia; ^3^Department of Internal Medicine, College of Medicine, Jouf University, Sakaka, Saudi Arabia

## Abstract

Pregnancy-related cardiovascular disease with stroke remains a considerable source of higher maternal morbidity and mortality occurs in periods of pregnancy, delivery, and postpartum. It is essential to counsel the mother before pregnancy by an expert cardiologist and obstetric team to discuss any event related to preexistent cardiac or past preeclampsia for estimation of maternal and fetal risks. In pregnancy, the cardiac state includes hypertensive disorders, ischemic heart disease, valvular disease, and postpartum stroke. The incidence of stroke is increasing in pregnancy, particularly in postpartum, and its strong relationship with hypertensive disorders of pregnancy (preeclampsia). The combined cardiologist and obstetrics team requires during pregnancy mainly due to the approach to the management of a cardiac disease that subsequently prevents stroke postpartum. Therefore, a general perception of cardiac disease during pregnancy, delivery, and postpartum should be a core knowledge extent for all cardiovascular and clinicians. Many studies provided linked that deregulation of microRNAs (miRNAs) in maternal circulation and placenta tissue may development of pregnancy complications including preeclampsia considered a diagnostic marker. The desire of this review provides a detailed outline of current knowledge and dealing in this field with strength on the physiological changes during pregnancy.

## 1. Introduction

Directly or indirectly pregnancy-related cardiovascular disease (CVD) is considered a leading cause of maternal death in both developed and developing countries [1, 2]. In the last few decades, a greater number of postpartum hospitalization and maternal death notified considering cardiovascular disease. Globally, around 1%-4% of cardiovascular diseases may complicate pregnancy [3]. It has been evidenced that nearly 0.2–0.4% of CVD is affected among all pregnant women [4]. Several studies accounted that progressively growing pregnancy with CVD, because of the increased number of women with congenital heart disease, a certain combination in their reproductive age, and advancing maternal age which includes risk factors as multifetal pregnancies, hypertension, hypertensive disorders of pregnancy (HDP; gestational hypertension and preeclampsia), and diabetes mellitus [5, 6]. Besides, ischemic heart disease (IHD), among acute myocardial infarction (AMI), is an indirect cause of maternal death associated with CVD in western countries [7]. In developed countries, the data suggested that the type of CVD in pregnancy is commonly related to valvular heart disease, in both congenital and acquired [8]. Conversely, it has been demonstrated that hypertensive disorders of pregnancy (HDP) women have a greater risk of stroke long-term, and approximately 50% of pregnancy strokes are associated with preeclampsia or eclampsia [9–11]. Pregnancy stroke is considered a dangerous period because prolonged disability following pregnancy around 7.7% results in maternal death [10]. Preeclampsia women have notified the chance of risk increased 2-fold development of stroke in their later life [11]. In 2011, a combination of the American Heart Association (AHA) and American Stroke Association reported that hypertensive disorder during pregnancy is considered a key risk factor for developing of cardiovascular diseases and advised to clinicians for routinely screen women who have a past of these complications [6]. It has been demonstrated that the expression of circulating miRNA altered during pregnancy is exaggerated by cardiac complications. In the past few years, the circulating miRNA become grown as a biomarker of diagnostic and prognosis value of cardiac failure [12], while a large number of studies published relations of miRNA with cardiac disease including preeclampsia, gestational hypertension, and postpartum cardiomyopathy. However, the present study reviews the miRNA and pregnancy-associated heart disease and stroke.

## 2. Cardiovascular Adaptations to Normal Pregnancy

**Figure 1:**
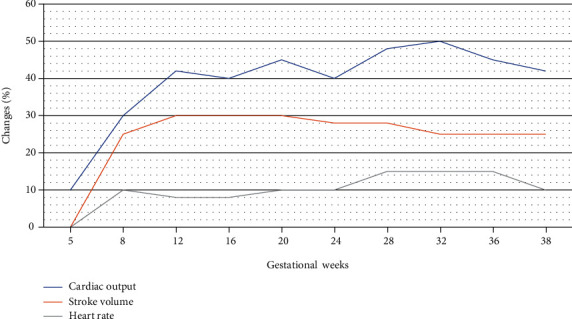
Physiological change in pregnancy.

Pregnancy altered the physiological function of the cardiovascular system by raising the metabolic demand and assurance uteroplacental adequate circulation for proper growth and development of the fetus. The preliminary physiologic change has seen lower systemic and pulmonary vascular resistance that subsequently lowers mean arterial blood pressure [13]. Therefore, a 30%-50% increase in the circulating plasma volume at 12 gestational weeks is a peak at the time of the third trimester. Meanwhile, heart rate increased by around 10 beats per minute [14]. Consequently, these changes may responsible for raising cardiac output which is shown in Figure 1, although the peak level of cardiac output detected in the third trimester of pregnancy was nearly above 50% at prepregnancy levels and a 25% consistent increase in stroke volume [15]. Additionally, a further 80% of cardiac output increased during delivery due to rapid and significant hemodynamic changes in the mother. However, with either vaginal or cesarean delivery, the hemodynamics returns to baseline approximately 2-4 weeks later.

## 3. Pregnancy in Advanced Maternal Age Impact on Cardiovascular Disease

**Figure 2:**
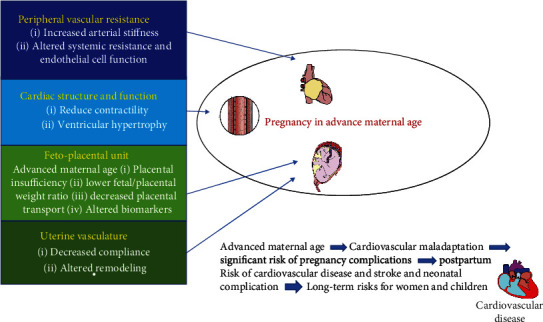
Advance maternal age has an impact on cardiac adaptation in pregnancy considered to have cardiac complications.

In the aging state, the biological function diminishes which subsequently affects the function of the endothelium and increases the activities of the sympathetic nervous system, subsequently leading to arterial stiffness and hypertension [16–18]. Hence, the advanced maternal aged cardiovascular system decreases the adaptation capacity to the vascular system during a physiological alteration in pregnancy. Unfortunately, there is insufficient data available in the research field about the relationship between advanced maternal age and the function of the vascular system in pregnancy. Meanwhile, some studies' evidence of the perception of vascular adaptation may be exaggerated by maternal age [19]. Data from survey analysis on 884 healthy pregnant women the ages ranging from 15 to 45 years detected a significant association between mean uterine artery resistance (as measured with pulsatility index (PI)) and maternal age, who is over the age of 35 years [19]; therefore, it is proposed that uterine artery resistances over advanced maternal age may have potential adverse effect on outcomes of pregnancy. On the other hand, the placenta is important for fetal growth and development; inadequate placental function may oppose effects on pregnancy outcomes including preeclampsia and stillbirth. Therefore, it is suggested that pregnancy at maternal age may be responsible for cardiac complications during pregnancy and also postpartum which is shown in Figure 2.

## 4. Cardiac Events and Stroke in Pregnancy

**Figure 3:**
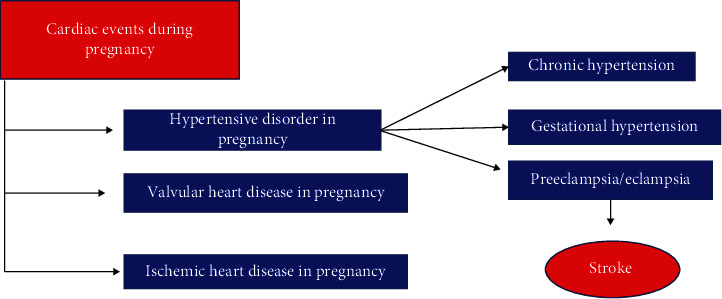
Flowchart of cardiac events during pregnancy.

Details are represented in a flowchart in Figure 3.

### 4.1. Hypertensive Disorders in Pregnancy

The delivery is approximately 912 per 10000 with hypertensive pregnant mothers estimated as a common disorder according to hospital data in the United States. According to the American College of Obstetricians and Gynecologists (ACOG), HDP is divided into four categories such as chronic hypertension, gestational hypertension, preeclampsia/eclampsia, and hypertension with superimposed preeclampsia [20]. However, preeclampsia/eclampsia significantly raises mortality in both mother and fetal. Preeclampsia is expressed when raising systolic blood pressure ≥ 140 mmHg or diastolic blood pressure ≥ 90 mmHg after 20 weeks of pregnancy with no previous history of hypertension. These circumstances usually affect approximately 2% to 8% of all pregnancies [21]. It has been reported that preeclampsia is subsequently 71%, 2.5-fold, and 4-fold risk for death of cardiovascular disease, coronary artery disease, and heart failure, respectively. Several studies evidence that regular exercise, diet, modified lifestyle, and cessation of smoking may reduce the risk of CVD and also prevent preeclampsia which was also suggested by both ACOG and AHA [20], although many studies recommended a low dose of aspirin in case of preterm birth at <34 weeks, late first trimester, chronic hypertension, and diabetes mellitus, which subsequently diminishes the danger of maternal myocardial ischemia, heart failure, and stroke. Conversely, selected drugs depend on the severity of hypertension including nifedipine, methyldopa, and labetalol that are essential for less-severe hypertension as considered first-line antihypertensive drugs, while labetalol and hydralazine are intravenously recommended for severe hypertension. Moreover, it is demanding antihypertensive therapy for pregnant mothers with HDP and continues postpartum for around 1-2 weeks.

### 4.2. Ischemic Heart Disease in Pregnancy (IHD)

Pregnancy with IHD is unlikely to develop but potentially dangerous state. Acute myocardial infarction has a 3- to 4-fold risk during pregnancy, while some studies evidence that per 100000 deliveries with IHD, about 2.8 to 8.1% may lead to an increase in the death rate by approximately 4.5% to 7.3% [22]. Several studies reported that acute myocardial infarction in pregnancy most probably causes myocardial infarction along nonobstructive coronary and spontaneous dissection of a coronary artery during pregnancy that is mostly occurring in the third trimester and postpartum period [23]. The treatment of IHD depends on patients' clinical presentation which is organized by the multidisciplinary team. According to a survey in 2016 in the United Kingdom, maternal mortality is the highest cause of ischemic heart disease (IHD) among one-fifth of heart disease with pregnancy being higher related to older age around 40 years or over. It is demonstrated during pregnancy, acute myocardial infraction is a risk that was compared with nonpregnant in the same age group [14]. In the United Kingdom, the obstetrics survey on one hundred fifty pregnant women identified ST-segment elevation MI (STEMI) as most commonly found compared to non-ST-segment elevation MI (NSTEMI) around the third trimester or postpartum showing approximately one-fifth of heart disease around the country. Most of the studies reviewed IHD in pregnancy in developed countries and identified the women with AMI due to one of the risk factors responsible for 40% older than 35 years, and parous women in developing countries showed smoking and reproductive age being common in nearly 15% [24]. A similar report was found in a study on 100 pregnant women with MI and 45% due to smoking causes. The principal management is the same for both pregnant and nonpregnant conditions which are represented in diagram 1. Besides, many studies recommended combination teams including obstetricians, cardiologists, obstetric anesthetists, and neonatal specializing in management from early to end of delivery. Low doses of aspirin below 150 mg/day are safe during pregnancy, while there was no extensive report about clopidogrel in pregnancy but few report evidence of no fetal toxicity produced after using clopidogrel during pregnancy. Additionally, primary percutaneous coronary intervention was done with stent implantation treatment for STEMI, although some researchers suggested using lead shielding to decrease the exposure to radiation when performing coronary angiography on the fetus. Conversely, several medications are considered safe during pregnancy and lactation, which also prevent further secondary cardiovascular disease (Table 1).

### 4.3. Valvular Heart Disease in Pregnancy

In the developing country, 80% development of valvular heart disease and 50% causes of maternal death during pregnancy, rheumatic fever is the main cause of these that are seen the first time during pregnancy [7]. Generally, in normal cardiac function, the regurgitant lesions are well tolerated during pregnancy, and the stenotic lesion is considered at higher risk of decompensation during pregnancy due to limited increase of cardiac output leading to increase gradients of transvalvular and upstream lesions pressure that subsequently decreases the tolerated in pregnancy [25]. However, rheumatic mitral stenosis is poorly tolerated in the pregnancy state which is the main fact for higher maternal mortality of cardiac causes in developing countries. The symptoms of mitral stenosis (MS) in the pregnant state present exertional dyspnoea and as well postural symptoms such as paroxysmal nocturnal dyspnoea and orthopnea. In the case of pulmonary edema, the middiastolic rumbling murmur is difficult to recognize at the level of apex, while left atrium enlargement and bifid P wave are essential to be detected by radiology and ECG investigations, respectively. Conversely, pulmonary edema is a risk during pregnancy because vascular resistance increases in the last trimester leading to a high filling pressure on the left side. Additionally, this risk further occurs during labor and postpartum immediately due to hyperdynamic circulation and blood volume increase at the third stage of labor. The principal management depends on the severity of MS; if moderate stenosis (valve area 1.0–1.5 cm^2^) was detected, then either regular close monitoring or intervention is required. In the case of asymptomatic and severe mitral stenosis (valve area < 1.0 cm^2^), the best method is elective percutaneous balloon valvuloplasty performed during the midtrimester or 20 weeks earlier of gestation [26]. However, open-heart surgery is ready for maternal life-threatening conditions. In pregnancy state, aortic stenosis (AS) is very rare, and asymptomatic AS during pregnancy is well tolerated though it is a severe condition. It is suggested that pregnancy may continue in the asymptomatic state when left ventricular size, function, and exercise test results are normal. Besides, the management of persistent symptoms of AS during pregnancy is percutaneous valvuloplasty. However, the treatment protocol for life-threatening conditions is the replacement of a valve followed by an early cesarean section. Also, for severe symptomatic AS, the delivery should be performed by cesarean section. Furthermore, aortic regurgitation (AR) mainly due to congenital bicuspid aortic valve, rheumatic heart disease, and prior endocarditis may result in functional regurgitation [27]. In pregnancy with AR, diuresis is the choice of medicine with nitrates and hydralazine for reduction of afterload; besides, close monitoring after delivery is needed due to dramatic changes of hemodynamics that subsequently develop pulmonary edema and respiratory distress [8]. Mitral valve prolapses and rheumatic heart disease are responsible for the development of mitral regurgitation (MR) in pregnancy, and MR is tolerated in pregnancy when left ventricular function is normal [8]. Medicine therapy like diuresis and afterload reduction and close monitoring are needed to follow delivery to avoid developing pulmonary edema.

### 4.4. Stroke in Pregnancy

**Figure 4:**
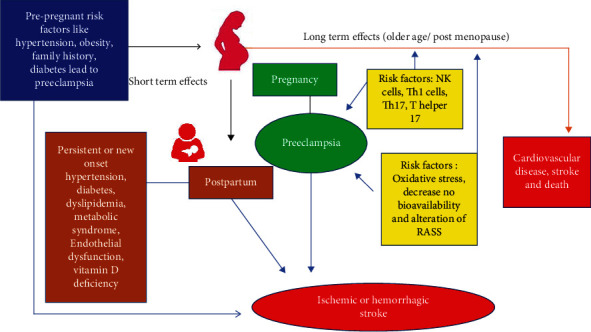
Long-term and short-term effects of a pregnant mother with cardiac disease and stroke.

Stroke is a distinct unaware impairment of neurological function through the neuronal injury from a vascular incident that is one of the major leading causes of disability and death. In developed countries, stroke (ischemic stroke (IS) and hemorrhagic stroke (HS)) may be overwhelmingly complicated during pregnancy and increases maternal mortality and morbidity [28]. According to the survey data in the United States, approximately 7.4% of maternal death by a pregnancy-related stroke were subsequently found to increase in Canada by nearly 60%. It has been evidenced that stroke-related pregnancy is the major cause of hypertensive disorder in pregnancy which represent 6.8% of maternal death in the US [29]; meanwhile, 40% to 70% of maternal death are due to preeclampsia which subsequently develop stroke as shown in Figure 4. Another study describes that 36% of pregnancy-associated strokes (PASs) occur as major causes of preeclampsia, and this preeclampsia later has 6-fold higher risk of stroke during puerperium [30]. Therefore, pregnancy-related stroke with preeclampsia may increase maternal mortality. Several studies reviewed and suggested that stroke is one of the risk factors during postpartum who has a history of either preeclampsia or eclampsia [31]. The pathophysiology and risk factors of preeclampsia and stroke are similar to hypertension, hypercoagulability, endothelial dysfunction, and dyslipidemia in addition cerebral vasomotor reactivity. The review of 31 pregnant women with stroke examined shows that 47% are ischemic strokes with preeclampsia and 43% are hemorrhagic strokes with preeclampsia. According to data from the United States, ischemic stroke and hemorrhagic stroke combined are detected as a risk in 30 per 100000 pregnancies [20]. However, it is established that hemorrhagic stroke is considered a common stroke found during pregnancy and postpartum associated with preeclampsia/eclampsia.

**Figure 5:**
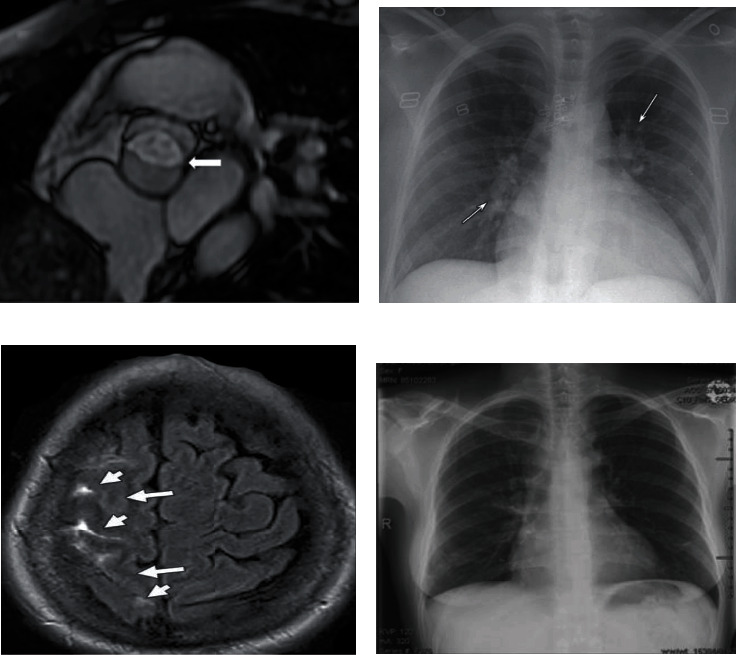
(a) In a woman, in 18 weeks of her 2nd pregnancy, the magnetic resonance appears bicuspid aortic valve (arrow) showing moderately enlarged ascending aorta up to 4.75 cm [28]. (b) Posteroanterior chest radiographs in a 22-year-old woman during and post pregnancy. At 36 weeks, the first radiograph was taken which showed an enlarged cardiac silhouette and engorged pulmonary vasculature (arrows) with apical redistribution [29]. (c) This is a case of a 24-week pregnant woman at her 30-year-old along subarachnoid hemorrhage due to eclampsia complication. Axial FLAIR MR image shows the presence of subarachnoid blood in the right posterior frontal and parietal sulci (short arrows) and subtle edema covering the underlying cortex (long arrows) [30]. (d) Peripartum cardiomyopathy showed in a pregnant woman by chest radiography [31].

Arterial ischemic stroke is an emergency condition and should be started with initial management several studies' recommendation; the management of stroke in pregnancy has similar to nonpregnant women as represented in Table 1 [29]. However, the common imaging finding is shown in Table 2 [32–35]. Several studies safely performed chest X-ray during pregnancy which is represented in Figure 5.

## 5. miRNAs and Pregnancy-Related Cardiac Disease with Stroke

**Figure 6:**
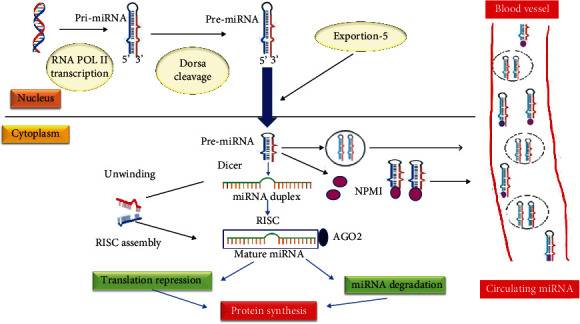
Mechanism of action of circulating miRNAs.

The miRNAs are noncoding RNA molecules, single-stranded with the family of short (19–25 nucleotides long); it is elaborate regulation of posttranscriptional gene through degrading mRNA or blocking its translation [36]. The genome is approximately 75% and 2% transcribed into RNA and gene with protein code, respectively; therefore, they preserve the normal cell function including cellular differentiation, proliferation, and apoptosis (Figure 6); bedside deregulations of these miRNAs may develop many diseases including preeclampsia in pregnancy. It has been documented the variable expression of miRNA in preeclamptic women which is represented in Table 3. The placenta, circulation, and umbilical vein show a specific pattern of miRNA and highly express MiR-141, miR-23a, miR-136, and some novel miRNA in the placenta [37]. The deregulations of miRNA indicate of disease condition, the higher miR-499a-5p expression in cerebrovascular and cardiovascular disease which is detected in the placenta of pregnancy-induced hypertension such as gestational hypertension and preeclampsia, whereas lower 26a-5p, miR-103a-3p, and miR-145-5p expressions are detected in the initial stage of preeclampsia (PE). However, the downregulated of the miR-548c-5p was detected in serum placental mononuclear cells in preeclampsia patients [38]. Consequently, these miRNAs act as a risk to increasing enduring cardiac disease in patients with preeclampsia. Therefore, the circulating miRNA in preeclampsia in pregnant women may consider a biomarker for initial recognition of diagnosis, monitoring, and follow-up preeclampsia during pregnancy. A previous study reported the upregulation of miR-210-3p and miR 210-5p was not only detected in preeclampsia, but also, miR-210 is present in pregnant heart failure patients [39]. There was deficiency data available for miRNA with stroke in pregnancy, but several studies reported miRNA is relevant for diagnosis, prognosis, and therapeutic role of stroke. In present study reviews, similar deregulations of miRNA in stroke and preeclampsia in pregnancy which are represented in Table 3 [37, 40].

## 6. Pregnancy Outcomes

The multidisciplinary team may improve the management of heart disease during pregnancy; they should be more concerned during physiological changes in pregnancy in a woman who has a history of heart disease to avoid both maternal and fetal mortality and morbidity. The management protocol should be done before the conception and must be conducted by a collaborative team including an obstetrician and a cardiologist. It has been accounted that maternal cardiac disease causes fetal uteroplacental insufficiently due to inadequate maternal cardiac output [41]. The study reviewed six hundred forty-six pregnant women with vulvar disease including mitral stenosis, aortic stenosis, mitral regurgitation, and aortic regurgitation with reported 6% and 5% stillbirth, 6%, 4%, 6%, and 1% death, and 2 miscarriages, respectively [15]. Conversely, the survey study on ischemic heart disease (IHD) reported fetal and neonatal complications including 32% premature, 4% abortion, 1% neonatal death, 10% intrauterine growth restriction (IUGR), 28% low birth weight baby (LBW), 1% fetal hemorrhage, and 5% acute respiratory distress syndrome (ARDS) [42]. Several studies observe the pregnancy outcome in a woman with heart disease and found 20% attack adverse effects of neonates on the survey in Canada, according to Cardiac Disease in Pregnancy (CARPREG) [38]; besides, the multinational Registry of Pregnancy and Cardiac Disease (ROPAC) presented increase fetal and neonatal mortality and morbidity. The study of hypertensive disease with stroke during pregnancy found roughly fetal and neonatal complications including intrauterine growth restriction (IUGR), preterm delivery, prediabetes, and prehypertension that may lead to causes of increased mortality and morbidity [43–45].

## 7. Conclusions

Pregnancy-associated cardiovascular disease is a potential long-term consequence risk for maternal and fetal health. The general hemodynamic, hormonal, and metabolic variations during pregnancy state a significant physiological burden on the cardiovascular system. The physiological changes during pregnancy and postpartum predominate the progress of cardiovascular events, and exacerbation of preexisting cardiac disease may contribute to a higher maternal mortality rate. During pregnancy, the diversity of hypertension disease is made parallel the risk for stroke and cardiac disease in future life. These days, the miRNA biomarker is used as a marker of noninvasive, diagnostic, and prognostic for early exposure to pregnancy-related cardiac disease with its complications. Preconception counseling and early participation of a multidisciplinary team are indispensable for the effective management of these complications.

## Figures and Tables

**Table 1:** Management strategies of cardiac disease and stroke in pregnancy.

Hypertensive disorder of pregnancy (HDP)	Ischemic heart disease in pregnancy (IHD)	Vulvar heart disease in pregnancy	Stroke in pregnancy
Precounseling to avoid pregnancy until control of hypertension or during pregnancy advise following below	Prepregnant council to delay pregnancy after treatment of IHD. If unexpected pregnant, then the following management	Pregnancy should be avoided in severe mitral and aortic valve disease. If unexpected pregnant, then the following management	Precounseling to control hypertension who has a previous history of preeclampsia

Investigation			
Urine test for proteinuria.	Positive stress ECG, MRI, and exercise testing evidence for IHD and recommendation coronary angiography, if clinically indicated.	Clinical and ECG knowledge are required sequentially for a pregnant woman to know the condition of valvular heart disease. The percutaneous valve intervention is the best treatment for those who are not responding to the medical therapy.	MRI images are considered an optimal modality during pregnancy, in case of missing timely taken MRI, then angiography CT and also perfusion CT can be chosen as a guide for proper interventional therapies.
Ophthalmoscopic examination.			
Blood values			
USG			

Risk factors			
Hypertension, obesity, and family history of diabetes	Hypertension and preeclampsia are strongly associated with AMI.	Rheumatic fever is a most common	Preeclampsia, eclampsia
			Mechanical heart valves

Pregnancy management			
Close follow-up	Close follow-up	Close follow-up	Closely monitoring. Common drugs used: labetalol, atenolol, methyldopa, nifedipine, warfarin, and heparin (low molecular weight), and direct oral anticoagulants.
Medical therapy: Nifedipine, methyldopa, labetalol, and hydralazine	Medical therapy: antiplatelet therapy, nitrates, beta-blockers, inotropes, and oxygen Intervention: PCI and cardiac surgery	Medical therapy for heart failure or arrhythmias Balloon valvuloplasty or surgical valve replacement	

Delivery			
Vaginal delivery with control of hypertension	Normal vaginal delivery unless cardiac and obstetrician indication. Continuous maternal cardiac monitoring. Continuous electronic fetal monitoring. Emergency cesarean section prior to cardiac surgery if needed.	If possible, vaginal delivery is preferred. Cesarean section is chosen when there is risk to the mother or fetus. Early delivery for clinical and hemodynamic worsening.	Vaginal delivery is the best approach if there is no obstetric contraindication.
Emergency cesarean section if required			

Complication			
Stroke, hypertension, and cardiac disease are responsible for the development of preeclampsia or eclampsia during pregnancy and also in postpartum	Cardiac arrest, heart failure, and ventricular tachycardia	Pulmonary edema, atrial arrhythmias, stroke, and heart failure	Reversible cerebral vasoconstriction syndrome can cause both ischemic and hemorrhagic stroke and the risk of cerebral venous sinus thrombosis (CVST). Long-term disability

Follow-up			
The utility of subclinical vascular measurements, such as cerebral or peripheral vasomotor reactivity, carotid intimal medial thickness, coronary calcification, or clinical and biochemical biomarkers, is needed to identify women with a history of preeclampsia at increased risk of future stroke.	Maternal cardiac monitoring for at least 48 hours after delivery	Hemodynamic monitoring at least 24 hours postpartum	Counseling and cardiovascular screening of women who have a past history of preeclampsia. As well as correction of the other vascular risk factors.

**Table 2:** The imaging modalities in pregnancy.

Imagines modality	Imaging finding	Indications
Electrocardiography	The interpretation of ECG the heart rotates to the left with a 15–20° leftward axis deviation in most women in pregnancy.	Finding structural heart diseases
Echocardiography	Usually, some changes occur in echo parameters during pregnancy such as an increase in valve gradient, changes in the thickness of the LV wall, and dilation of the chamber quite mild.	Delivery of the status for the structures and functions of the heart
Chest radiography	Diagnostic cardiovascular radiographic examination	The condition of the heart, lungs, airways, blood vessels, and the bones of the spine and chest showing by images
Computed tomography	The X-rays reveal the images of the part of the body obtained in the various orthogonal plane.	Anomalous coronary artery and aortic dissection
Magnetic resonance imaging	Any abnormality of fetus	Congenital heart disease, aortic disease, and stroke
	Cerebral edema, ischemia, and hemorrhage	
Ultrasonography	Fetal heart rate, fetal position, and fetal presentation	The initial procedure for monitoring fetal well-being in pregnancy

**Table 3:** The different expressions of the miRNA in preeclampsia and stroke.

MicroRNAs	Preeclampsia	Source	Stroke	Source
miR-145	↑	Human maternal plasma	↑	Tissue/rat
miR-21	↑	Human maternal plasma	↑	Tissue/rat
miR-210	↑	Human placenta	↓	Human blood
miR-26a	↑	Human maternal plasma	↓	Tissue, blood/rat
miR-26b	↑	Human maternal plasma	↓	Tissue, blood/rat
miR-328	↓	Human placenta	↓	Tissue, blood/rat
miR-29b	↑	Primary human umbilical vein endothelial cells	↑	Tissue, blood/rat
miR-204	↑	Human placenta	↑	Tissue/rat
miR-23a	↑	Human maternal plasma	↑	Tissue, blood/rat
miR-335	↑	Human placenta	↓	Tissue/rat
miR-150	↓	Human placenta	↑	Tissue, blood/rat
miR-126	↓	Human placenta	↓	Human blood
miR-155	↑	Human placenta	↓	Tissue, blood/rat
miR-451	↓	Human maternal plasma	↑	Tissue, blood/rat
miR-107	↓	Human maternal plasma	↑	Tissue, blood/rat
miR-185	↓	Human maternal plasma	↑	Tissue, blood/rat
